# Preliminary validation of a web-based MRI scoring system for children with chronic nonbacterial osteomyelitis (ChRonic nonbacterial Osteomyelitis Magnetic Resonance Imaging Scoring: CROMRIS)

**DOI:** 10.1186/s12969-025-01135-x

**Published:** 2025-08-01

**Authors:** Farzana Nuruzzaman, T. Shawn Sato, Jennifer Stimec, Ramesh S. Iyer, Andrew Carbert, Joel Paschke, Lauren Potts, Meinrad Beer, Ming Huang, Johanna Monsalve, Anh-Vu Ngo, Mahesh Thapa, Xiaoyue Zhang, Walter P. Maksymowych, Polly J. Ferguson, Yongdong Zhao, For the CARRA CRMO Workgroup

**Affiliations:** 1 Renaissance School of Medicine at Stony Brook University, Stony Brook, New York USA; 2 University of Iowa Carver College of Medicine, Iowa City, Iowa USA; 3 The Hospital for Sick Children, Toronto, Ontario Canada; 4 University of Washington, Seattle, Washington USA; 5 CARE Arthritis LTD, Edmonton, Alberta Canada; 6 Illustrator/Patient Research Partner, Long Beach, California USA; 7 University Hospital Ulm, Ulm, Germany; 8 Mount Sinai Hospital, New York, New York USA; 9 University of Alberta, Edmonton, Alberta Canada; 10 Seattle Children’s Hospital, Seattle, Washington USA

**Keywords:** Chronic recurrent multifocal osteomyelitis, Pediatric, Scoring method, Magnetic resonance imaging

## Abstract

**Background:**

The ChRonic nonbacterial Osteomyelitis Magnetic Resonance Imaging Scoring (CROMRIS) tool was developed to assess specific characteristics of bone and soft tissue inflammation on MR images of patients with CNO; however, this tool was labor intensive to utilize. We aimed (1) to refine and adapt this scoring method, (2) to assess the usability of this web-based CROMRIS system among radiologists and (3) to evaluate the absolute agreement of the components and summary CROMRIS scores at each body site, and the interrater reliability.

**Methods:**

We used a qualitative, user-centered design approach involving software developers, rheumatologists, radiologists, and a patient artist to adapt the paper-based scoring system to a web-based prototype that was further refined by monthly meetings between the group members. A clickable-schematic-based CROMRIS system was developed to include all body regions: head (skull/mandible), spine, torso (clavicle, sternum, and ribs), pelvis, hands, feet, arms, and legs. Readers scored individual bone units to indicate the presence of bone marrow hyperintensity on STIR images (score 0–1), soft tissue/periosteal hyperintensity of surrounding tissue (score 0–1), and bony expansion (score 0–1), and quantified the signal size of the CNO lesion (scores 1–3 defined as < 25%, 25–50%, or > 50% of the estimated volume, respectively). The sum of these parameters for lesions detected on fluid-sensitive sequences was the CROMIS Activity Index (maximum score 720). Feedback for usability was reported with descriptive content analysis and continuous variables as means and categorical variables as percentages. Interrater reliability was assessed by free-marginal kappa (k) statistics and the intraclass correlation coefficient (ICC).

**Results:**

The mean system usability score increased from 64.5 (below average) to 75 (above average) after user feedback. Interrater reliability for the CROMRIS Activity Index was excellent for clavicle, tibia, cervical and lumbar spines (> 0.9) and good to moderate for the remainder of the body regions. The mean kappa of each category of bones was > 0.6 demonstrating substantial interrater reliability among radiologists for the bone sites most affected by CNO, namely the long bones and clavicle.

**Conclusion:**

The web-based CROMRIS portal developed was usable and showed substantial-moderate agreement in the total CROMRIS Activity Index total scores among experienced radiologists after self-guided learning of the atlas and video. This tool can potentially be used in future clinical trials after calibration.

**Supplementary Information:**

The online version contains supplementary material available at 10.1186/s12969-025-01135-x.

## Introduction

Chronic nonbacterial osteomyelitis (CNO) is a rare autoinflammatory bone disease that causes skeletal inflammation characterized by bone pain and swelling that primarily affects children [1]. Unfortunately, no known clinical or laboratory markers reliably measure disease activity in response to treatment. Pediatric rheumatologists often use imaging as a marker of disease activity [2]. Whole-body magnetic resonance imaging (WBMRI) specifically has many benefits that emphasize the variable appearance of the lesions including the ability to detect subclinical lesions and identify typical distributions of bone involvement. MR imaging is also free of radiation and is used to assess the response to treatment. However, imaging interpretation is primarily descriptive and subject to interrater variability [3–5].

We aimed to standardize the radiological interpretation of MRI in CNO by creating the Chronic Nonbacterial Magnetic Resonance Imaging Scoring (CROMRIS) tool to assess specific characteristics of bone and soft tissue inflammation as well as skeletal damage [6]. This radiologist-based comprehensive scoring tool demonstrated sensitivity to changes after aggressive treatment in a retrospective cohort study as well as acceptable interrater reliability in previous studies [6].The CROMRIS was simplified by Capponi et al. to create a radiological activity index (RAI) [7]. Despite these advancements, the practical use of this scoring system was limited by being paper-based and thus labor intensive to use and prone to human errors. Therefore, in partnership with CARE Arthritis LTD (CARE Arthritis), an academic research organization in Canada, we have developed a web based CROMRIS system with clickable schematic diagrams and the ability to directly input scores with simplified binary scoring to improve feasibility for use in future research trials in CNO. In this report, we describe the validation of this platform in terms of feasibility and reliability.

## Methods

### Platform development and usability testing

**Fig. 1:**
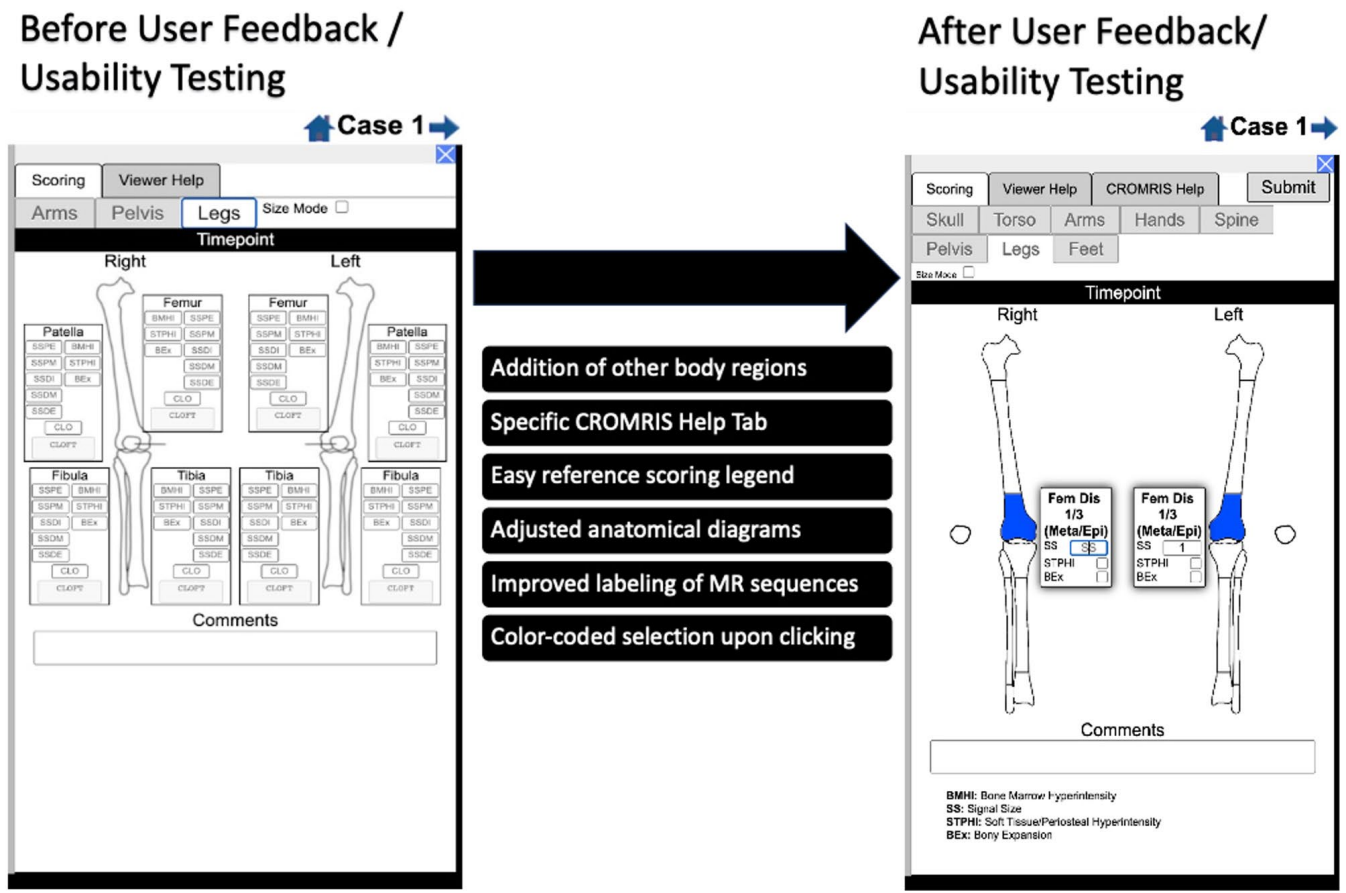
Screenshot of web-based CROMRIS portal on tab for long bones of lower extremity before and after feedback sessions/usability testing. Legend: BMHI: Bone Marrow Hyperintensity; SS: Signal Size; STPHI: Soft Tissue/Periosteal Hyperintensity; BEx: Bony Expansion; Fem Dis 1/3 Meta: distal one-third femur (metaphysis/epiphysis)

The CARRA CRMO Working Group used CARE Arthritis’s online medical image viewer and integrated scoring interface for imaging-based scoring systems to develop a prototype web-based CROMRIS tool limited to the arms and legs [8–10]. Monthly meetings between software developers, pediatric rheumatologists, pediatric radiologists, and an illustrator led to a beta version that included the entire skeleton. A purposive sample of radiologists (*n* = 7) provided feedback on the beta version in a demo session on April 11, 2022 via semi structured surveys including the General Usability Testing Survey and System Usability Scale (SUS) (Stony Brook University #IRB2021-00033). Iterative changes to the platform were made based on responses and further discussion between May 2022 and December 2022 (Fig. 1). The goal of the project was to create a usable web-based scoring tool for MRI in CNO (based on the ISO 9241 standard), defined as meeting the following criteria:

- Effective - The user can completely and reliably interpret and score an MR image in CNO.
- Efficient - The user spends less time and effort making navigation and action choices than manual paper-based scoring/interpretation of CNO MR images.
- Engaging - The visual design of the web-based interface is appealing and user-friendly.
- Easy to Learn - The web-based CROMRIS interface builds upon the user’s prior knowledge of the paper-based CROMRIS and uses the same terminology, designs elements, and controls as the previous version. It can also be used by individuals not familiar with CROMRIS.
- Accessible - Available on any Internet connected device to be used in different institutions world-wide.

We developed a comprehensive usable online portal for scoring of radiological disease activity in CNO available on the CARE Arthritis website (https://www.carearthritis.com/mriportal/crmo/index/*)*, accounts free to registered academic users) [11]. The entire skeleton is divided into scoring tabs: Skull, Torso, Arms, Hands, Spine, Pelvis, Legs, and Feet. Within each tab, the region was subdivided into the following *bone units* defined as individual scorable regions determined by the developers:

- Head: skull (L, R), maxilla (L, R), mandible (L, R).
- Torso: clavicles (L, R), scapula (L, R), ribs 1–12 (L, R), sternum.
- Arms: humerus, radius, ulna divided into proximal, middle, and distal 1/3 (L, R).
- Hands: phalanges (L, R), metacarpals (L, R), carpals (L, R).
- Spine: C1-7, T1-12, L1-5.
- Pelvis: periacetabulum (L, R), ilium (L, R), pubis/superior pubic ramus (L, R), ischium/inferior pubic ramus (L, R), and sacroiliac joints (L, R).
- Legs: femur, tibia, fibula divided into proximal, middle, and distal thirds (L, R), patella (L, R).
- Feet: forefoot (L, R), midfoot (L, R), hindfoot (L, R).

**Fig. 2:**
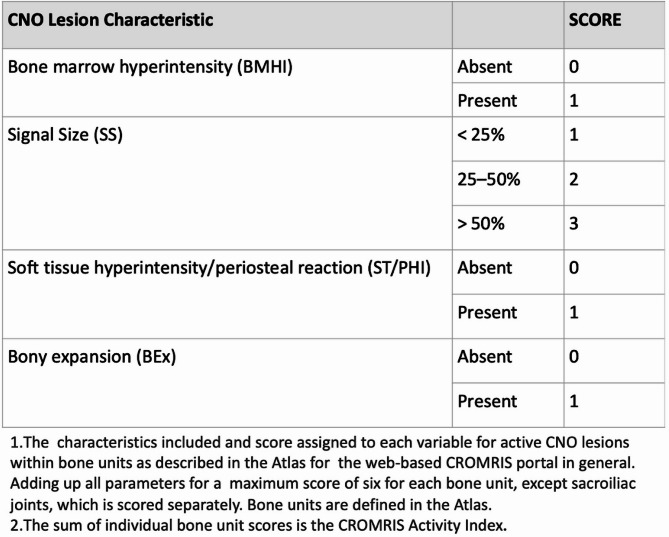
Scoring of active chronic nonbacterial osteomyelitis (CNO) lesions in general bone units in the web-based CROMRIS portal. Legend: CNO: chronic nonbacterial osteomyelitis

**Fig. 3:**
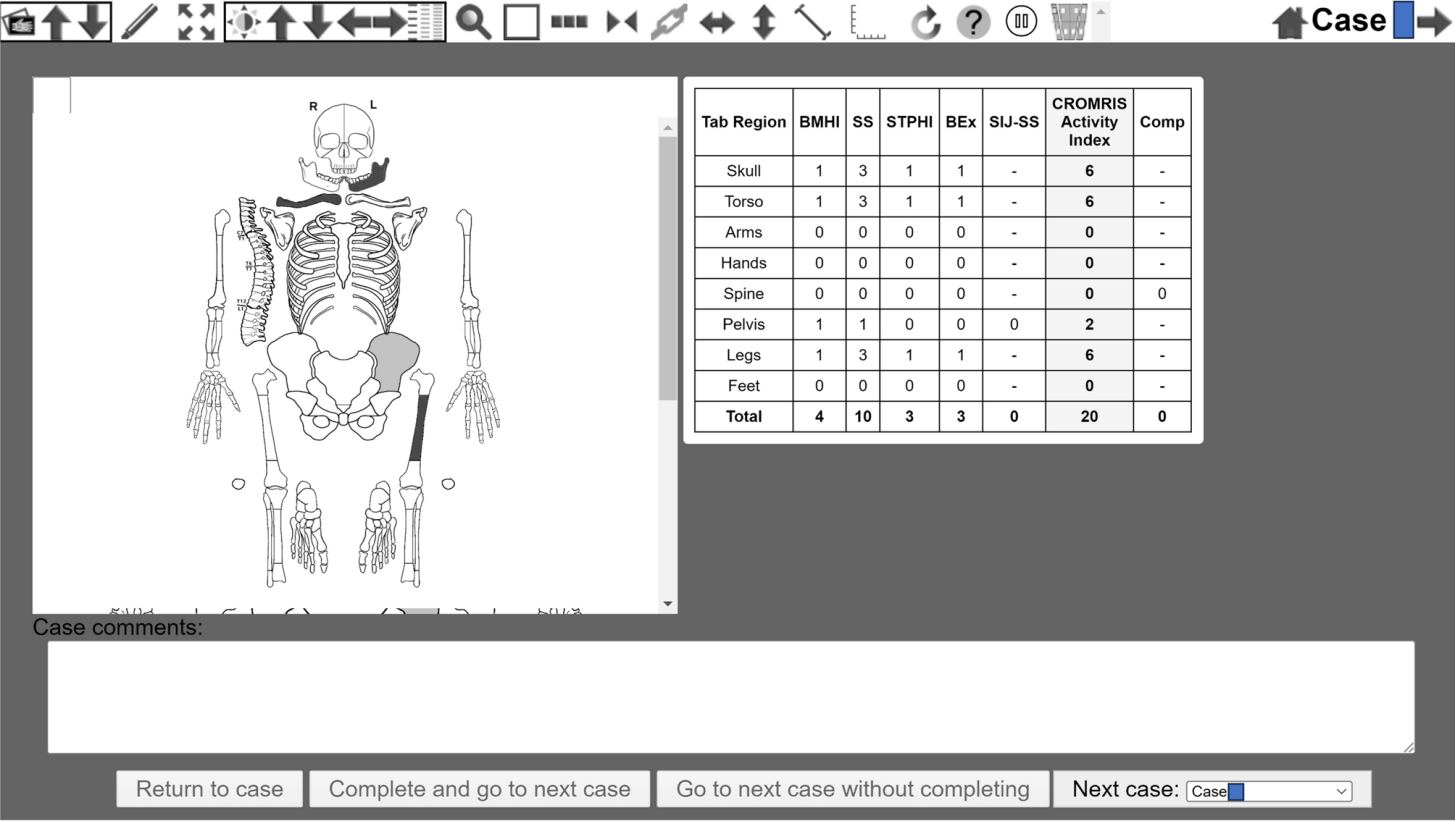
Screenshot of summary page for the final web-based CROMRIS portal for a sample Case. Legend: BMHI: Bone Marrow Hyperintensity; SS: Signal Size; STPHI: Soft Tissue/Periosteal Hyperintensity; BEx: Bony Expansion; SIJ-SS: Sacroiliac Joint Signal Size; Comp: vertebral compression

The scoring of bone units as seen on Fig. 2 is accomplished by touch-clicking on the scoring interface to indicate the presence/absence (yes/no) of the following variables: (1) bone marrow hyperintensity on fluid-sensitive sequences (STIR, fat saturation or TIRM) (score 0–1); (2) soft tissue/periosteal hyperintensity of surrounding tissue or presence of abnormal signal intensity other than a normal luminal structure (i.e., bladder, intestine, cerebrospinal fluid space, vasculature) within surrounding tissue on a fluid-sensitive sequence (score 0–1) and (3) bony expansion, or an enlarged bone contour that is greater than expected (score 0–1, no/yes). Numerical scores for the extent of high signal intensity within each bone marrow unit are also completed according to a grading scheme (“1” defined as < 25% of estimated volume, “2” defined as 25–50%; and “3” defined as > 50% of the estimated volume). The maximum activity score for most bone units is 6. The minimum score for each identified lesion is 2 (i.e. sum of “1” or the presence of bone marrow hyperintensity and “1” as a measurement of signal size < 25% of estimated volume). The presence of vertebral compression is also indicated for each individual vertebra (score 0–1, yes/no). Each sacroiliac joint is divided into 4 quadrants: upper sacral, upper iliac, lower iliac, lower sacral (“1” if 1 quadrant involved, “2” defined as 2 quadrants involved; and “3” defined as 3 *or* 4 quadrants involved), with a maximum score of 3 for each SI joint. The total score from all active CNO lesions was summed up as the final CROMRIS Activity Index on a final output page with an associated composite image after each study with darker color intensity of blue correlating with a larger signal size. For example, a lesion with a signal size of “3” is the darkest shade of blue on the final schematic diagram on the scoring summary page of the web-based platform. A lesion with a signal size of “1” is the lightest shade of blue. Upon scoring completion, the Web tool outputs a spreadsheet file containing scores of 0/1 per region and summary statistics (Fig. 3). The CROMRIS activity index score across 119 bone units (and two SIJ units) ranges from 0 to 720. Vertebral compression is not included in the summary score of the CROMRIS Activity Index as it is a sign of damage, rather than disease activity in CNO. However, one can see the total number of vertebral compressions in the final summary tab after the study is completely scored. Joint effusion and measures of damage/complications, such as non-CNO bony abnormalities, limb hypertrophy, kyphosis, bony expansion without bone edema and pathological fractures were excluded from the index developed here.

### Atlas and training video

We provided readers for self-guided study with an instructional slide presentation (36 slides) including a scoring atlas giving examples of true CNO lesions and a 13 min video demonstrating CROMIS scoring on the CARE Arthritis website (https://www.carearthritis.com/mriportal/crmo/index/*).* The tutorial is also available on CARE Arthritis’s YouTube Channel to expand accessibility (https://www.youtube.com/watch?v=zDrDreKCgrY).

### Reliability module

A total of twenty-nine sets of pre-existing whole-body MRI (WBMRI) scans were used for assessing reliability. The study was approved by the institutional review boards from Iowa Children’s Hospital (# 201609778) and Seattle Children’s Hospital (#2683). Healthy controls were included in the analyses to ensure variability in the sample. The WB scans were composed of STIR images of the skull (mandible), torso (clavicle, ribs), upper extremities (arms, hands), pelvis, lower extremities (legs, feet).

The studies were acquired either on a 1.5-T (Avanto; Siemens Medical Solutions, USA, Inc., Malvern, PA) or 3-T (Siemens Prisma Fit) magnet, with scan times approximately 45 min per patient. Three fellowship-trained pediatric radiologists (TSS, RSI, and JS), all with > 10 years of experience and with dedicated clinical time regularly to interpret WB-MRI studies in patients with CNO, independently reviewed and scored these cases directly on the web platform and were blinded to the diagnosis. Prior to scoring, the readers reviewed the online tutorial as well as a comprehensive atlas for scoring instructions Each reader did not score more than 10 cases per day to avoid visual fatigue.

### Data analysis

Usability was assessed in two phases (in May 2022 and in December 2022) via the System Usability Scale (SUS), which is a Likert scale in which respondents indicate their level of agreement or disagreement on a scale of 1 to 5 for 10 statements [12]. (Supplementary File 1). The SUS has been widely used in the evaluation of a range of systems, and this has led to normative data. Raw SUS scores are converted into percentile ranks where 68% is considered the cutoff for an instrument likely to be widely applied (i.e. usable). Feedback was reported by descriptive analysis, with continuous variables as the means and categorical variables as percentages.

Descriptive analysis was performed to assess the prevalence of abnormalities among 29 cases at each body site. For each case, each rater evaluated 121 body units. In this study, we aggregated scores at the body site level by summing the scores of individual scorable bone units. For the extremities or bones on both sides of the body, the left and right scores were combined. Regions of aggregated scores and included individual scorable bone units are as follows: *arms* (humerus, radius, ulna), *feet* (forefoot, midfoot, hindfoot), *hands* (phalanges, metacarpals, carpals), *legs* (femur, tibia, fibula, patella), *pelvis* (sacroiliac joints, other pelvis sites), *skull* (maxilla, mandible, other skull), *spine* (cervical vertebrae, thoracic vertebrae, lumbar vertebrae), and *torso* (sternum, ribs, scapulae, clavicles).

Abnormality at each body site was defined as a score > 2 assigned by at least 2 out of the 3 raters for that body site (i.e. the raters scored “1” for BMHI and at least a “1” for a signal size for a lesion). For specific variables, namely bone marrow hyperintensity (HI), signal size, soft tissue/periosteal HI, bony expansion, spinal compression, the absolute agreement was defined as all the raters assigning the exact same score. For CROMRIS summary score, if all the pairwise comparisons between any 2 raters are ≤ 2, an absolute agreement is reached. Interrater reliability for abnormal signal and severity were assessed using free-marginal kappa (k) statistics. The kappa result was interpreted as suggested by Cohen [12]: values ≤ 0 as indicated no agreement and 0.01–0.20 indicated no to slight agreement, 0.21–0.40 indicated fair agreement, 0.41 – 0.60 indicated moderate agreement, 0.61–0.80 indicated substantial agreement, and 0.81–1.00 indicated almost perfect agreement [13]. The intraclass coefficient agreement (ICC) values of CROMRIS Activity scores across 3 raters were measured for each body region separately. The ICCs are presented as absolute agreement for the individual reader pairs and for all readers together (overall ICC). An ICC value of < 0.4 was designated fair; ≥ 0.4 but < 0.6 moderate; ≥ 0.6 but < 0.8 good; ≥ 0.8 but < 0.9 very good; and ≥ 0.9 excellent. Analysis was performed using SAS 9.4 [14].

We also compared the self-reported time to score on paper-based CROMRIS scoring form to the mean time to score each case by readers of our reliability module on the web-based platform to determine the feasibility of the broad use of this tool.

## Results

A clickable-schematic-based CROMRIS was developed after usability feedback sessions to include all body regions: head (skull/mandible), spine, torso (clavicle, sternum, ribs), pelvis, hands, feet, arms, and legs [11]. The components of the scoring are shown in figure. Some notable changes incorporated into the web-based portal after feedback sessions with radiologists included: adjusting the rotation of hand diagrams to match MRI rotation; adding labels to individual vertebrae of the spine diagram for ease of clicking on the correct bone unit; adding direct access to the atlas/scoring instructions on the portal and adding color selection upon clicking on a bone unit with a darker shade of blue indicating a higher signal size score (Fig. 2). After each case, there is a summary output page with a table that sums the scores including the CROMRIS Activity Index and a composite schematic as a visual representation of the CNO lesions (Fig. 3). Visual factors (i.e. font size, color scheme) and anatomical diagrams were among the features “liked best” by the survey respondents. The mean system usability scores increased from 64.5 (below average) to 75 (above average) after the feedback sessions. All respondents agreed that the web-based CROMRIS was “easy to learn” and that the “various functions of the web-based CROMRIS were well integrated.”

**Fig. 4:**
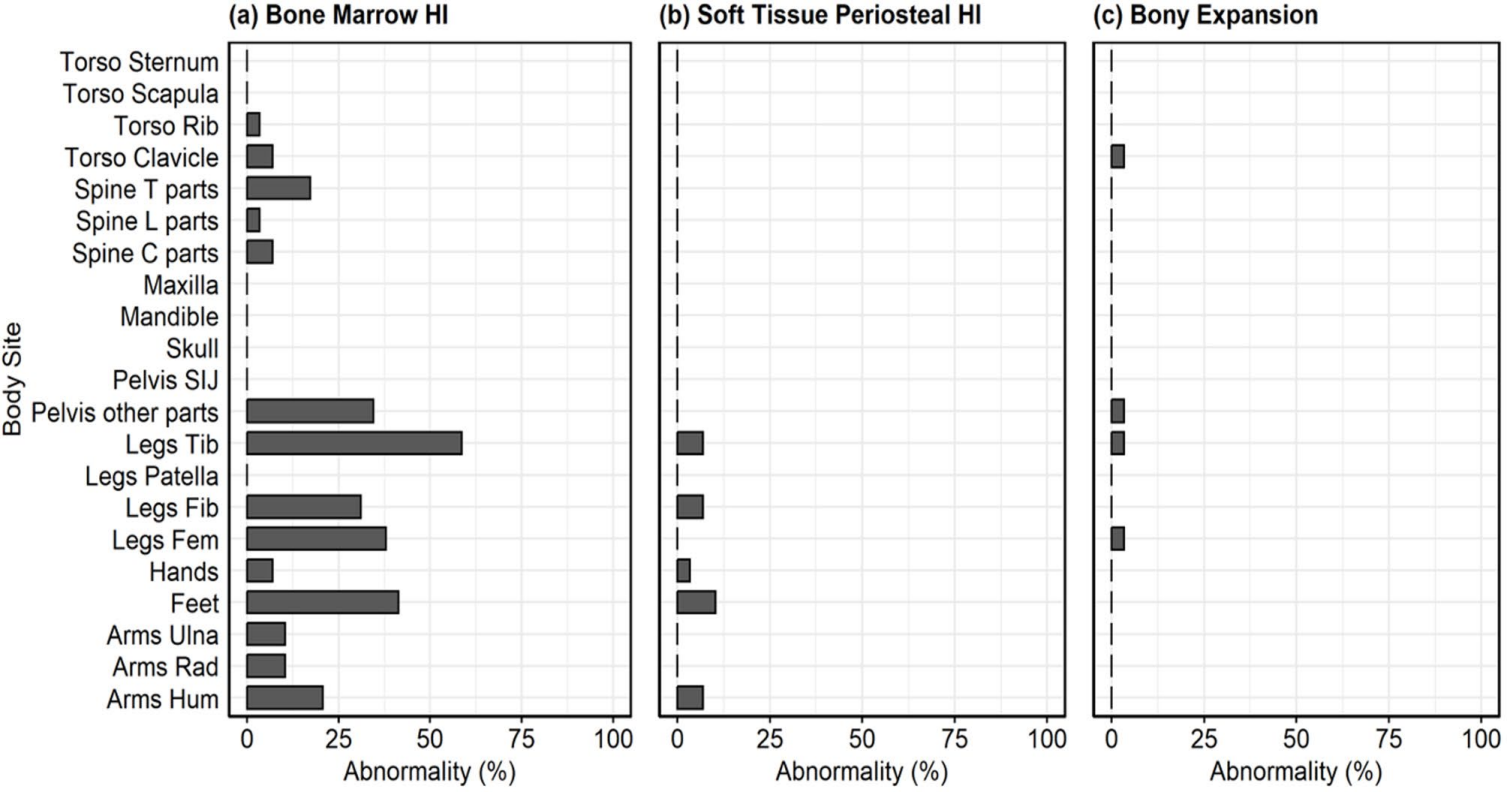
Prevalence of abnormalities across 2 raters at each body site based on bone marrow hyperintensity (HI), soft tissue/periosteal HI, and bony expansion scores among 29 cases. HI: hyperintensity; Spine T parts: thoracic spine; Spine L parts: lumbar spine; Spine C parts: cervical spine; SIJ: sacroiliac joint; Tib: tibia; Fib: fibula; Fem: femur; Rad: radius; Hum: humerus. BMHI: Bone Marrow Hyperintensity; SS: Signal Size; STPHI: Soft Tissue/Periosteal Hyperintensity; BEx: Bony Expansion

**Fig. 5:**
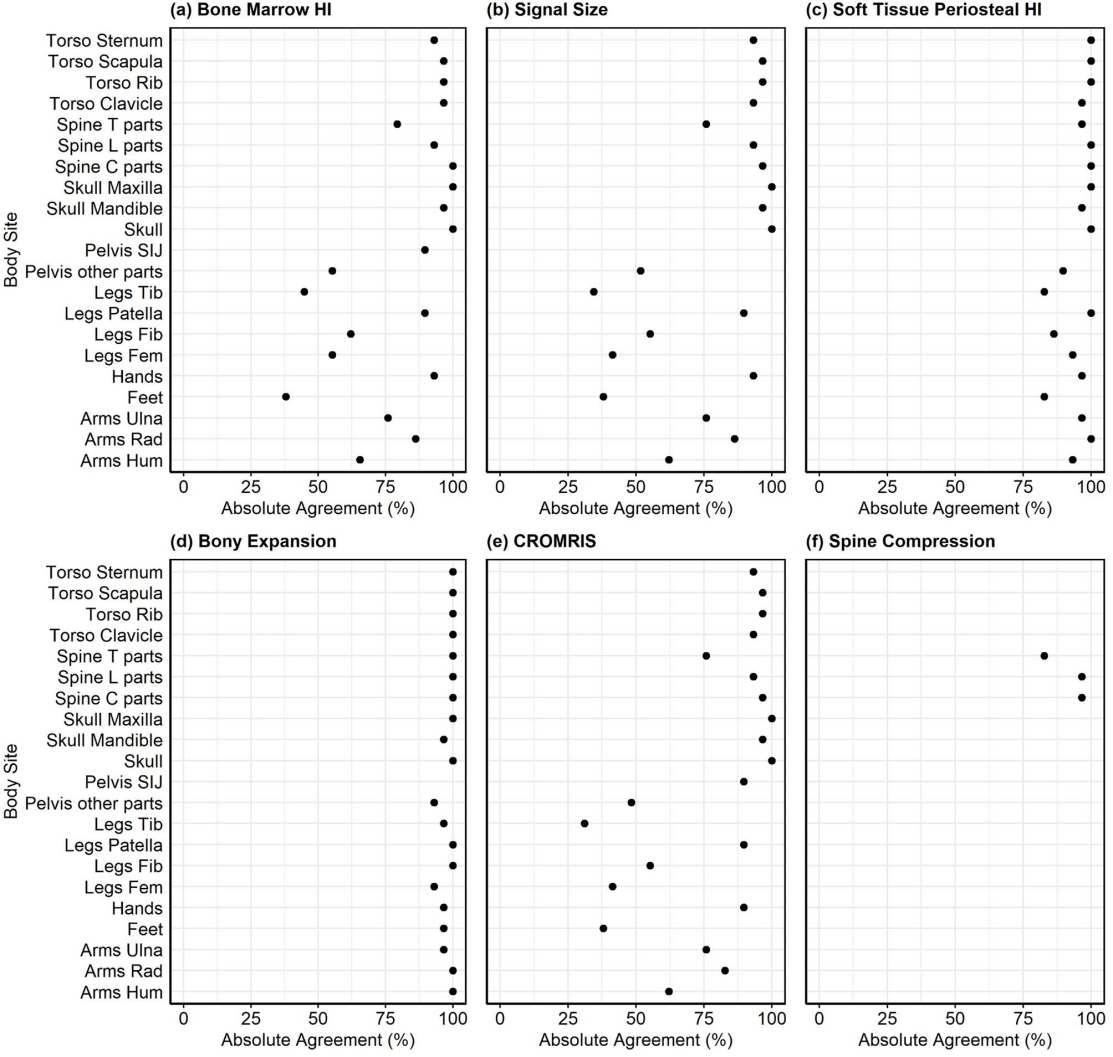
Absolute agreement across 3 raters at each body site among 29 cases. Legend: HI: hyperintensity; Spine T parts: thoracic spine; Spine L parts: lumbar spine; Spine C parts: cervical spine; SIJ: sacroiliac joint; Tib: tibia; Fib: fibula; Fem: femur; Rad: radius; Hum: humerus

Among all bone sites, the prevalence of abnormalities was the highest at the tibia (59%), feet (41%), femur (38%), and pelvis (34%) across 2 raters (Fig. 4). There were no abnormalities identified by the raters in the skull, scapula, sternum, and patella in this module. Absolute agreement of CROMRIS Activity Index scores across the 3 raters was greater than 70% in most bone sites, except the tibia, femur, and feet, which was correlated with areas of highest prevalence of CNO lesions (Fig. 5e).

**Fig. 6:**
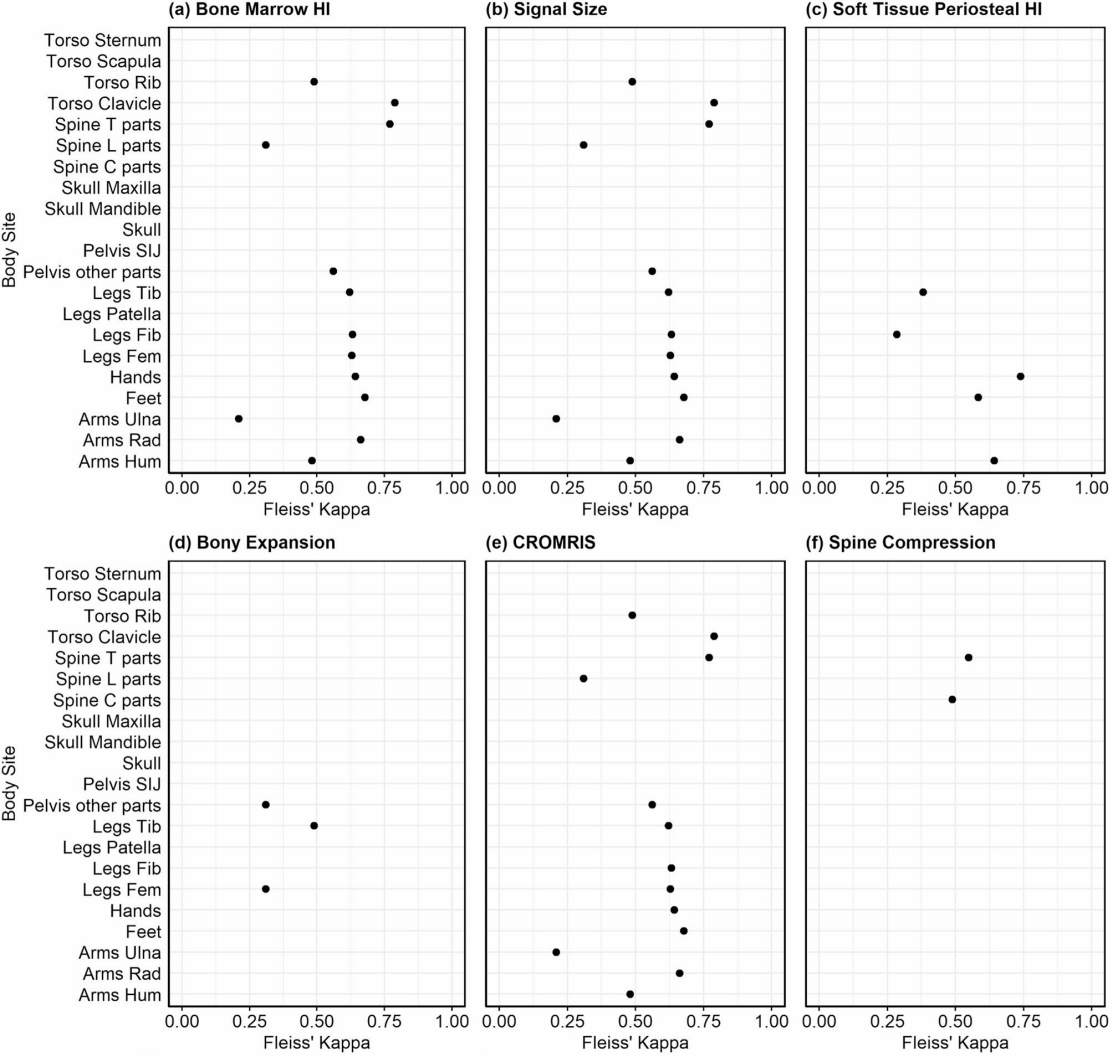
Interrater agreement across 3 raters at each body site based on Fleiss’ Kappa. Legend: HI: hyperintensity; Spine T parts: thoracic spine; Spine L parts: lumbar spine; Spine C parts: cervical spine; SIJ: sacroiliac joint; Tib: tibia; Fib: fibula; Fem: femur; Rad: radius; Hum: humerus

The mean kappa coefficient of each category of bones was near or > 0.5 demonstrating moderate to substantial interrater agreement for CROMRIS summary scores among radiologists for most bone sites (Fig. 6), except lumbar spine, ulna, patella, mandible, sternum, scapula, and sacroiliac joints, which had mean kappa scores ranging from − 0.04 to 0.21. ICC scores of CROMRIS Activity index were excellent for tibia, clavicle, cervical spine, thoracic spine (ICC 0.9–0.99). ICC scores for humerus, radius, feet, hands, femur, fibula, ribs, pelvis, lumbar spine were in the good to moderate range (ICC 0.67–0.89). As all the 3 raters gave score 0 for all the 29 cases for maxilla and skull, the ICC agreement score is not evaluable for these sites. ICC scores for the mandible and patella were low, likely due lack of variability of the scores in the cases or small number of cases including lesions in those areas.

In contrast, the mean kappa coefficient of each category of bones was > 0.7 with majority > 0.9 demonstrating substantial/almost perfect reliability of CROMRIS in the study by Zhao et al. [6]. The differences in interrater reliability and agreement from the manual version previously reported by Zhao et al., may be related to variations in way the scorable bone units were combined and analyzed between the two studies (i.e. left and right sides were combined in this study) or due to differences between patients’ lesion frequency between the two studies. Regardless, the online-based platform is more accessible for users around the world with fewer opportunities for errors that result from manual transcriptions of scores and more ease of scoring, making this a valuable tool.

The average time spent to score each case on the web-based platform was 8.4 min (SD 5.2). This is more than half the amount of mean time to score WBMRI cases on the paper-based scoring report as indicated on the usability surveys by the sample of pediatric radiologist readers (19 min (SD 15.9)). As time constraints are often cited as a barrier to participation in research [15], the efficiency of this tool can lead to broader and more equitable participation from sites around the world.

## Discussion

This web-based CROMRIS portal is a usable and accessible semiquantitative tool to standardize the assessment of MR imaging in children with CNO. There was moderate agreement of CROMRIS Activity Index total scores amongst experienced radiologists after self-guided learning of the atlas and video. The quantification of CNO lesions is essential for assessing treatment response, especially at the spine and pelvis, where the proportion of absolution agreement of CROMRIS Activity Index across 3 raters was greater than 75% in the spine and pelvis. The identification of lesions in the spine is particularly critical because of the potential long-term morbidity associated with inaccurate reporting of spinal lesions. Similarly, identification of CNO lesions in the pelvis is also critical as it may reduce the need for invasive biopsies when the differential diagnosis of malignancy is of concern.

A critical gap in the successful execution of clinical trials for CNO which can provide evidence-based treatment recommendations for CNO, is the lack of objective and validated outcome measures to assess radiological disease activity. While work to develop outcome measures is ongoing [16], MR imaging is practically used in the clinical setting to assess disease activity. The assessment of disease activity on MR for CNO has evolved over the years. In prior studies, CNO lesions on MRI were reported by an “osteitis score” consisting of the number of active lesions and their anatomical locations [17]. In patients with multiple sites of osteitis, the highest MRI score was used for the reference patient to determine treatment effect, which may not be accurate to assess the entire disease burden for the patient. The Radiologic Index for Nonbacterial Osteitis (RINBO) score, described by Arnoldi, generates a total score summing the score of the lesions and shows fair agreement with the clinical evaluation of each bone site [18]. A modified RINBO score described by Andreasan also included the maximal size of radiologically active lesions [19]. However, in both RINBO scoring and modified RINBO scoring, the maximal size of the lesions is in absolute terms, not as relative to the size of each bone. The use of relative size of lesion to the bone is more appropriate in growing children in which bones eventually increase in size through time.

### Study limitations

While human readers bias towards scoring a positive BMHI consistent with CNO in a reliability module, we attempted to decrease the false positive rate by providing negative controls in this module. Another limitation is that healthy controls were also not age-or-sex-matched in the MR images. While the amount of time spent scoring on this web-based platform was almost half the amount of time spent on the paper-based CROMRIS system, further studies are required to assess whether scoring timing is the same after a prespecified time frame. Another study limitation was that it did not include scoring pre- and post-treatment scans, though prior studies did suggest that the paper-based CROMRIS was shown to document change so one would expect similar results to a web-based CROMRIS [6].

## Conclusions

While this work has resulted in a productive tool, additional work needs to be done to calibrate readers to identify the minimum threshold of identification of the BMHI consistent with CNO. A consensus-based set of scans for reader calibration will be developed to enhance interrater reliability for clinical trials. It is also not known whether radiologically active lesions are equivalent to clinically significant lesions and additional studies are needed to answer this question. Regardless, we propose that the web-based CROMRIS will be an important and practical tool for measuring outcomes and allow further research that will bring meaningful changes in the care of patients with CNO.

## Electronic supplementary material

Below is the link to the electronic supplementary material.

Supplementary Material 1

## Data Availability

No datasets were generated or analysed during the current study.
