# Supplementary material for: Preliminary validation of a web-based MRI scoring system for children with chronic nonbacterial osteomyelitis (ChRonic nonbacterial Osteomyelitis Magnetic Resonance Imaging Scoring: CROMRIS)
Source: Pediatr Rheumatol Online J. 2025 Aug 1;23:85. doi: 10.1186/s12969-025-01135-x (PMC12317626; doi:10.1186/s12969-025-01135-x)
Supplement: Supplementary file 1 — Supplementary Material 1 [file 12969_2025_1135_MOESM1_ESM.docx]

Appendix A.

**System Usability Scale of System Evaluation of web-based CROMRIS tool**

**State your level of agreement with the following statements on a scale of 1-5.**

**(1) Strongly disagree; (2) Disagree; (3) Neither agree nor disagree; (4) Agree; (5) Strongly agree.**

1. I think that I would like to use the web-based CROMRIS scoring system more frequently than the paper-based CROMRIS scoring system
2. I found the web-based CROMRIS scoring system unnecessarily complex
3. I thought the web-based CROMRIS scoring system was easy to use.
4. I think that I would need the support of a technical person to be able to use web-based CROMRIS scoring system
5. I found the various functions in this web-based CROMRIS scoring system were well integrated.
6. I thought there was too much inconsistency in the web-based CROMRIS scoring system.
7. I would imagine that most radiologists would learn to use the web-based CROMRIS scoring system very quickly.
8. I found the web-based CROMRIS scoring system very cumbersome to use.
9. I felt very confident using the web-based CROMRIS scoring system.
10. I needed to learn a lot of things before I could get going with the web-based CROMRIS scoring system.
11. I think that I would like to use the web-based CROMRIS scoring system in future clinical trials.
12. I think web-based CROMRIS scoring system allows for faster turnaround time of scoring of WBMRI in CNO patients than prior manual versions.
13. I think the web-based CROMRIS scoring system allows for more accurate reporting of radiological interpretation of WBMRIs in CNO patients than prior manual version
